# Networked Behaviors Associated With a Large-Scale Secure Messaging Network: Cross-Sectional Secondary Data Analysis

**DOI:** 10.2196/66544

**Published:** 2025-07-10

**Authors:** Laura Rosa Baratta, Linlin Xia, Daphne Lew, Elise Eiden, Y Jasmine Wu, Noshir Contractor, Bruce L Lambert, Sunny S Lou, Thomas Kannampallil

**Affiliations:** 1 Division of Biology & Biomedical Sciences, Washington University School of Medicine in St. Louis, St. Louis, MO, United States; 2 Division of Computational & Data Sciences, Washington University in St. Louis, St. Louis, MO, United States; 3 Institute for Informatics, Data Science & Biostatistics (I2DB), Washington University School of Medicine in St. Louis, 660 South Euclid Avenue, Campus Box 8054, St. Louis, MO, 63110, United States, 1 314-273-7801; 4 Department of Anesthesiology, Washington University School of Medicine in St. Louis, St. Louis, MO, United States; 5 The Wharton School, University of Pennsylvania, Philadelphia, PA, United States; 6 Department of Industry Engineering and Management Science, Northwestern University, Evanston, IL, United States; 7 Department of Communication Studies, Northwestern University, Evanston, IL, United States; 8 Department of Computer Science and Engineering, Washington University in St. Louis, St. Louis, MO, United States

**Keywords:** communication, electronic health record, EHR, interprofessional communication, secure messaging, social network analysis, social network, interprofessional, messaging network, messaging, health care communication, message, network analysis, behavior, messaging platforms

## Abstract

**Background:**

Communication among health care professionals is essential for effective clinical care. Asynchronous text-based clinician communication—secure messaging—is rapidly becoming the preferred mode of communication. The use of secure messaging platforms across health care institutions creates large-scale communication networks that can be used to characterize how interaction structures affect the behaviors and outcomes of network members. However, the understanding of the structure and interactions within these networks is relatively limited.

**Objective:**

This study investigates the characteristics of a large-scale secure messaging network and its association with health care professional messaging behaviors.

**Methods:**

Data on electronic health record–integrated secure messaging use from 14 inpatient and 282 outpatient practice locations within a large Midwestern health system over a 6-month period (June 1, 2023, through November 30, 2023) were collected. Social network analysis techniques were used to quantify the global (network)- and node (health care professional)-level properties of the network. Hierarchical clustering techniques were used to identify clusters of health care professionals based on network characteristics; associations between the clusters and the following messaging behaviors were assessed: message read time, message response time, total volume of messages, character length of messages sent, and character length of messages received.

**Results:**

The dataset included 31,800 health care professionals and 7,672,832 messages; the resultant messaging network consisted of 31,800 nodes and 1,228,041 edges. Network characteristics differed based on practice location and professional roles (*P*<.001). Specifically, pharmacists and advanced practice providers, as well as those working in inpatient settings, had the highest values for all network metrics considered. Four clusters were identified, representing differences in connectivity within the network. Statistically significant differences across clusters were identified between all considered secure messaging behaviors (*P*<.001). One of the clusters with 1109 nodes, consisting mostly of physicians and other inpatient health care professionals, had the highest values for all node-level metrics compared to the other clusters found. This cluster also had the quickest message read and response times and handled the largest volume of messages per day.

**Conclusions:**

Secure messaging use within a large health care system manifested as an expansive communication network where connectivity varied based on a health care professional’s role and their practice setting. Furthermore, our findings highlighted a relationship between health care professionals’ connectivity in the network and their daily secure messaging behaviors. These findings provide insights into the complexities of communication and coordination structures among health care providers and downstream secure messaging use. Understanding how secure messaging is used among health care professionals can offer insights into interventions aimed at streamlining communication, which may, in turn, potentially enhance clinician work behaviors and patient outcomes.

## Introduction

Communication among health care professionals is key to effective clinical care, with reports suggesting that health care professionals spend >80% of their time on clinical communication [1,2]. Although face-to-face communication is often preferred, it is often not possible due to a lack of geographical proximity or other constraints [3]. As such, health care professionals rely on a number of synchronous (eg, phone) or asynchronous (eg, secure messaging, email, and pager) modes of communication. Asynchronous text-based communication—generally referred to as secure messaging—can be used either within the electronic health record (EHR) or through independent secure mobile platforms and is rapidly becoming the primary mode of communication in modern clinical settings [4]. Although recent studies have described the use of secure messaging in large health systems and its impact [5-10], our understanding of the structure and interactions within large clinical secure messaging networks is relatively limited.

Social network analysis methods allow for the quantitative and qualitative characterization of communication networks, including the behavioral patterns of use, interactions, spread of content or actions, and temporal changes in behaviors [11-13]. In other words, these approaches help in characterizing overall social interaction structure while simultaneously allowing for the investigation of how indirect connections, position within a network, and involvement of individual participants affect the behaviors, attitudes, and outcomes of other network members [14-22].

Prior studies using such methods for exploring interactive clinician-to-clinician communication have primarily relied on self-reported data (eg, surveys and interviews) using relatively small sample sizes of participants [23-31]. More recent studies have sought to characterize the communication behaviors of clinicians, often in specific patient populations (eg, patients with cancer) [32,33]. For example, Steitz et al [33] ascertained the communication network of care teams and characterized the message volume exchanged regarding breast cancer patients.

In contrast to prior studies, this study aims to understand the structure of secure messaging behaviors among health care professionals from a social network perspective. The primary aims of this study are (1) to characterize the network properties of a large-scale EHR-integrated secure messaging network and (2) to determine the association between network properties (eg, connectivity) and health care professionals’ messaging behaviors.

## Methods

### Study Setting

This was a cross-sectional study conducted at 14 inpatient hospitals and 282 outpatient clinics affiliated with BJC HealthCare and Washington University School of Medicine. Hospitals and clinics included both academic and community practice settings serving diverse rural, suburban, and urban populations across Missouri and Illinois. All hospitals and clinics used the same Epic EHR system (Epic Systems Corporation).

Epic’s Secure Chat virtual messaging platform is embedded within the EHR and allows health care professionals to send and receive messages. Messages are organized (ie, “threaded”) as conversations, and a patient identifier can be attached (as needed) to a conversation to facilitate direct patient chart access from the conversation. Secure Chat was launched across all study sites in September 2019 and is accessible through the desktop and mobile versions of Epic.

### Data Collection

Event logs related to secure messaging in dyadic conversations (ie, between pairs of health care professionals) using Epic’s Secure Chat were retrieved from Epic EHR’s Clarity database for the period June 1, 2023, through November 30, 2023. The choice of this period for the secure messaging data was primarily based on pragmatic considerations related to the availability of a continuous sample of data for at least 6 months, with no periods of missing data.

Communication event logs included time stamps recording when messages were sent and received, when messages were read (if they were), and when messages were responded to (if they were); the conversation thread to which a message belonged (ie, a conversation ID); the identities of the sender and receiver of the message; and the character length of each message. Additional metadata included both the senders’ and receivers’ most recent professional roles and most common practice locations. The content of the included messages was not retrieved or used for this study.

### Data Analysis

#### Data Categorization

The data contained a total of 74 unique health care professional roles, which were categorized into the following: physician, nurse, pharmacist, advanced practice provider (APP, ie, nurse practitioner or physician assistant), therapist (physical, occupational, or speech language), medical assistant or technician, social worker or case management, or other (as reported previously [5]). Practice locations were categorized as inpatient or outpatient.

#### Network Analysis

##### Developing the Messaging Network

We constructed a global secure messaging network of dyadic conversations. Each node represented a health care professional who sent at least 1 message during the study period (June 1, 2023, through November 30, 2023). Edges were constructed between individuals if at least one message was sent between them, and edge directionality was maintained to indicate the sender and the receiver. Pairs of nodes could have a maximum of 2 edges between them, representing reciprocated communication. Edge weights were assigned as the sum of the total character length sent among all messages by the sender to the corresponding receiver over the study period.

The giant connected component (GCC)—a maximum set of nodes such that each included node can be reached from any other node regardless of the direction of the edges [34]—was used for subsequent characterizations and analyses. We excluded nonconnected nodes since our study period is a cross-sectional snapshot in time, and it was very likely that nonconnected individuals are either remnants of closed conversations or the beginning conversations of a new user.

##### Network Characteristics

To illustrate the structural properties of the network, we generated node-level and global metrics. Node-level metrics represent the importance, social power, and control of information flow among nodes based on their connectivity within a network [35]. We included the following commonly used node-level network metrics (see detailed descriptions and examples in Table 1): indegree [36], outdegree [36], closeness [26,37-39], eigenvector [37,39,40], betweenness centrality [26,36-41], and local clustering coefficient [35,37,38]. These network metrics provide insights into how users participate and shape communication dynamics (see Table 1). For example, closeness centrality indicates users who can easily reach—or be reached by—others due to their positional proximity in the network to all other users; similarly, the clustering coefficient measures a tendency of a user’s contacts to also communicate with each other, revealing whether a user is communicating with mutually connected contacts.

**Table 1.** Definition and clinical implications for node-level network metrics.

Network metric	Definition	Clinical implications
Indegree	Number of incoming connections; edge weights were not considered.	The number of health care professionals a given health care professional sends messages to.
Outdegree	Number of outgoing connections; edge weights were not considered.	The number of health care professionals a given health care professional is receiving messages from.
Closeness centrality	The inverse of the sum of the lengths of the shortest paths, where a path is a sequence of nodes in which consecutive pairs of nonrepeating nodes are linked by an edge, to all other nodes. $\frac{N-1}{\sum_{y}d\left(y,x\right)}$ where *N* is the total number of nodes; *d*(*x*,*y*) is the length of the shortest, inverse-weighted path between *x* and *y*, where the weighted, shortest path is the minimum sum of weights for a given distance between nodes *x* and *y*.	Health care professionals with greater closeness have the shortest path lengths to all other users. Health care professionals with higher closeness can more quickly disseminate information throughout the entire network.
Eigenvector centrality	Proportional to the sum of the centralities of a given node’s neighbors. $x_{i}\frac{1}{\lambda }\sum_{j=1}^{n}a_{ij}x_{j}$ where *x* denotes the centrality of nodes *i* and *j,* and *a* represents whether an edge exists (*a*=edge weight) or not (*a*=0) between nodes *i* and *j*. Edge weights were considered.	A measure of the influence of a health care professional based on their connection to other well-connected individuals. A health care professional with a high eigenvector is centrally in direct communication with highly connected individuals in the network, and they have many connections to others. These individuals potentially receive a greater amount of incoming information from their incoming connections and can spread that information more efficiently through their outgoing connections.
Betweenness centrality	The number of shortest paths that pass through a given node. $\sum_{s,t\in \frac{V\left(G\right)}{v}}\frac{\sigma_{st}\left(v\right)}{\sigma_{st}}$ *σ*_*st*_ denotes the weighted shortest path, the minimum sum of the inverse of the edge weights, between nodes *s* and *t*, and *σ*_*st*_ (*v*) is the weighted shortest path through node *v*.	The extent to which a health care professional must act as a communication bridge or mediator between other health care professionals who are not directly connected. These individuals can act as connectors or brokers among groups of individuals who are not otherwise currently communicating.
Clustering coefficient	The fraction of pairs of nodes, which are neighbors of a given node, that are connected to each other by edges. Edge weights were not considered.	The tendency for a node to be embedded in a dense local neighborhood. Indicates the extent of clique structure for a given node.

Global network metrics help to understand the overall connectivity of the communication network (see detailed descriptions and examples in Table 2). Global metrics included the following: overall network density [40], reciprocity [26,35], diameter [36], transitivity [35,42], and assortativity [41]. These global-level measures illustrate the collective communication interactions to assess aspects such as the network’s cohesion, reachability, and structure. For example, the network density metric captures the proportion of connections relative to all possible connections in a network of a given size (total number of nodes), signifying the overall level of interactions; similarly, the diameter numerically indicates the longest shortest path between any two individuals, providing a sense of the greatest number of steps it takes to traverse the network (see Table 2).

**Table 2.** Definition and clinical implications for global-level network metrics.

Network metric	Definition	Clinical implications
Density	The ratio of actual connections to the possible connections. Edge weights were ignored.	Indicates how cohesive the communication network is. Higher density indicates a more cohesive communication network, showing that health care professionals are directly communicating with the majority of the other health care professionals within the network.
Reciprocity	The ratio of directional connections that are reciprocated. Edge weights were not considered.	Indicates whether health care professionals receive responses for messages they send out to other users. This indicates how mutual communication is between individuals in the network.
Diameter	The longest distance, where distance is the sum of the edge weight between the farthest nodes, to traverse the entire network. Edge weights were considered.	An indication of how expansive the network is in terms of the longest path consisting of communicating health care professionals.
Transitivity	The fraction of pairs of neighboring nodes of a given node that are connected to each other by edges. Edge weights were not considered.	The likelihood that two providers sharing a messaging partner are also messaging each other. This could signify that a network is composed of a lot of small cliques as opposed to wide-reaching communication cascades.
Assortativity	Determines the tendency of edges to connect similar nodes by calculating the fraction of edges that connect nodes of the same class for a given node attribute (eg, location, health care professional role, and total degree). Edge weights were not considered.	Quantifies whether nodes directly communicating with one another in a network generally share characteristics or differ.

##### K-Core Hierarchy

To explore the connective hierarchy of nodes within the global network, a k-core decomposition was constructed to categorize nodes into hierarchical bins. K-core decomposition is used to identify progressively central cores and discover the structural hierarchy of a network [43]. Previous studies have shown that identifying influential nodes using the k-core approach enables the discovery of central nodes that are highly influential in the transfer of information, in the contagion process, and in the resilience of the system [44,45].

In the secure messaging network, we performed a k-core decomposition based on the outdegree properties to identify the central (ie, influential) health care professionals. K-cores were based on the median and IQR of the outdegrees of the GCC such that the 25th percentile, median, and 75th percentile delineated the cores into 4 groups. Individual nodes were then sorted into each core depending on their outdegree and whether it was equal to or above the designated outdegree for that core. The k-core labeled as the first core had the health care professionals with the highest quartile of outdegrees, and the k-core labeled as the fourth core had individuals in the lowest quartile of outdegrees.

##### Clusters of Secure Messaging Health Care Professionals

To determine the association between a health care professional’s network characteristics and their behavioral messaging characteristics, we used a hierarchical clustering analysis to determine clusters based on network characteristics. We then clustered the health care professionals into clusters using node-level metrics (see Table 1). Features were standardized to a unit variance and a mean of zero to ensure consideration of all features as equally important in the clustering algorithm. To determine the optimal number of clusters, we calculated the silhouette score for values of 2 through 10 possible clusters.

##### Secure Messaging Behaviors

For each health care professional, we generated 4 measures related to secure messaging behaviors. Average read time for a message was calculated as the difference between a message’s sent timestamp and its read timestamp; average response time for a message was calculated as the time difference between the first message sent by a sender and the first message sent by the responder in a conversation. The average total volume of messages included the total of both sent and received messages for a health care professional per day. Finally, the average character length per message sent and received for each health care professional was calculated as the number of characters of the messages’ text including spaces but excluding trailing spaces.

### Statistical Analysis

Descriptive statistics for continuous metrics were calculated as medians and IQRs. Due to the nonnormal distribution of both the network and secure messaging metrics, nonparametric tests were used to test for statistical significance. Specifically, Mann-Whitney *U* tests were used to assess the differences in each numerical network metric between location setting categories (inpatient vs outpatient), the median differences were reported, and 95% CI were generated with bootstrapping for this comparison; Kruskal-Wallis tests were used to assess the differences in each network metric among the 8 health care professional role categories. Furthermore, chi-square tests were used to investigate whether the distribution of practice locations differed among the k-core hierarchy groups and for the distribution differences of health care professional roles per k-core. Finally, Kruskal-Wallis and a post hoc test, Wilcoxon rank-sum tests, were used to assess the differences between the secure messaging behaviors across clusters. Median differences and 95% CIs were reported for each comparison.

Data processing and clustering analysis were performed using Python 3.9.7 with the iGraph [46] and NetworkX [47] packages for network construction and network metric outputs and Scikit-learn [48] for hierarchical clustering. Statistical analyses were performed in R version 4.2.2 (R Foundation for Statistical Computing).

### Ethical Considerations

This study was approved by the Washington University institutional review board (IRB) with a waiver for informed consent (IRB #202205084). This was a retrospective study, and participants’ data were deidentified (eg, grouped based on IDs and roles) for analysis, and only trained researchers who were approved by the Washington University IRB office had access to the data. The participants were not compensated.

## Results

### Overall Messaging Patterns

This study included 31,800 health care professionals and 7,672,832 messages within 1,539,059 dyadic conversations. The included health care professionals practiced at 14 inpatient and 282 outpatient locations. Health care professionals had a median of 16 (IQR 4‐49) messaging partners over the study period.

The network included 31,800 nodes and 1,228,041 edges. The GCC comprised 29,808 nodes (93.7% of total nodes) and 1,225,307 edges (99.8% of total edges). The GCC had a low density of 0.00138 and a low transitivity of 0.09. The GCC diameter was 65,078. However, the reciprocity was high, 0.92, indicating most individuals received reciprocated responses to their messages. Assortativity over the GCC was found to be 0.044, 0.41, and 0.001 for the total degree (ie, the sum of indegree and outdegree), practice setting (ie, inpatient vs outpatient), and provider group (eg, pharmacists), respectively.

### Network Characteristics

#### Inpatient vs Outpatient Practice Setting

Health care professionals who practiced in an inpatient setting had significantly higher values (all *P*<.001) for the following network metrics compared with those who practiced in outpatient settings: eigenvector centrality (median –6.9e-11, 95% CI –1.0e-10 to –4.2e-11), betweenness centrality (median –174, 95% CI –359 to –72), outdegree (median 11, 95% CI 10‐11), and indegree (median 11, 95% CI 9‐11; Table 3). However, the local clustering coefficient and closeness were not statistically different between practice settings.

**Table 3.** Node-level properties stratified by location and clinical role.

	Eigenvector centrality, median (IQR)	Betweenness, median (IQR)	Clustering coefficient, median (IQR)	Outdegree, median (IQR)	Indegree, median (IQR)	Closeness, median (IQR)
Location						
Inpatient	1.6e-10 (6.6e-12 to 3.5e-09)	4 (0 to 59,612)	0.121 (0.049 to 0.231)	22 (5 to 60)	22 (6 to 60)	206 (156 to 241)
Outpatient	2.3e-10 (8.4e-12 to 4.3e-09)	178 (0 to 90,139)	0.120 (0.017 to 0.31)	11 (3 to 28)	11 (4 to 28)	207 (160 to 240)
Clinical role						
Advanced practice provider	6.8e-10 (3.7e-11 to 1.4e-08)	59,611 (0 to 301,500)	0.094 (0.043 to 0.19)	36 (9 to 102)	36 (10 to 107)	230 (191 to 254)
Medical assistant or technician	2.4e-11 (1.1e-12 to 4.9e-10)	0 (0 to 29,806)	0.150 (0 to 0.333)	7 (2 to 18)	7 (3 to 17)	176 (124 to 218)
Nurse	3.9e-10 (2e-11 to 5.7e-09)	54 (0 to 59,610)	0.147 (0.073 to 0.244)	26 (8 to 58)	27 (9 to 58)	218 (174 to 246)
Other	1.4e-12 (6.5e-15 to 5.5e-11)	0 (0 to 29,806)	0.134 (0 to 0.429)	4 (2 to 13)	5 (2 to 12)	136 (87 to 194)
Pharmacist	1.0e-09 (9.2e-11 to 1.2e-08)	31,331 (0 to 175,330)	0.110 (0.063 to 0.217)	53 (16 to 134)	47 (16 to 116)	229 (199 to 252)
Physician	9.2e-10 (7e-11 to 1.3e-08)	9701 (0 to 166,441)	0.068 (0.036 to 0.121)	24 (6 to 90)	27 (8 to 98)	221 (184 to 250)
Social work or case management	7.1e-10 (3.7e-11 to 9.6e-09)	36,015 (0 to 275,353)	0.144 (0.074 to 0.225)	33 (8 to 80)	32 (8 to 81)	229 (198 to 258)
Therapist	3.8e-11 (2.5e-12 to 6.7e-10)	0 (0 to 29,806)	0.143 (0.053 to 0.286)	14 (4 to 39)	15 (5 to 40)	195 (159 to 224)

**Figure 1.**
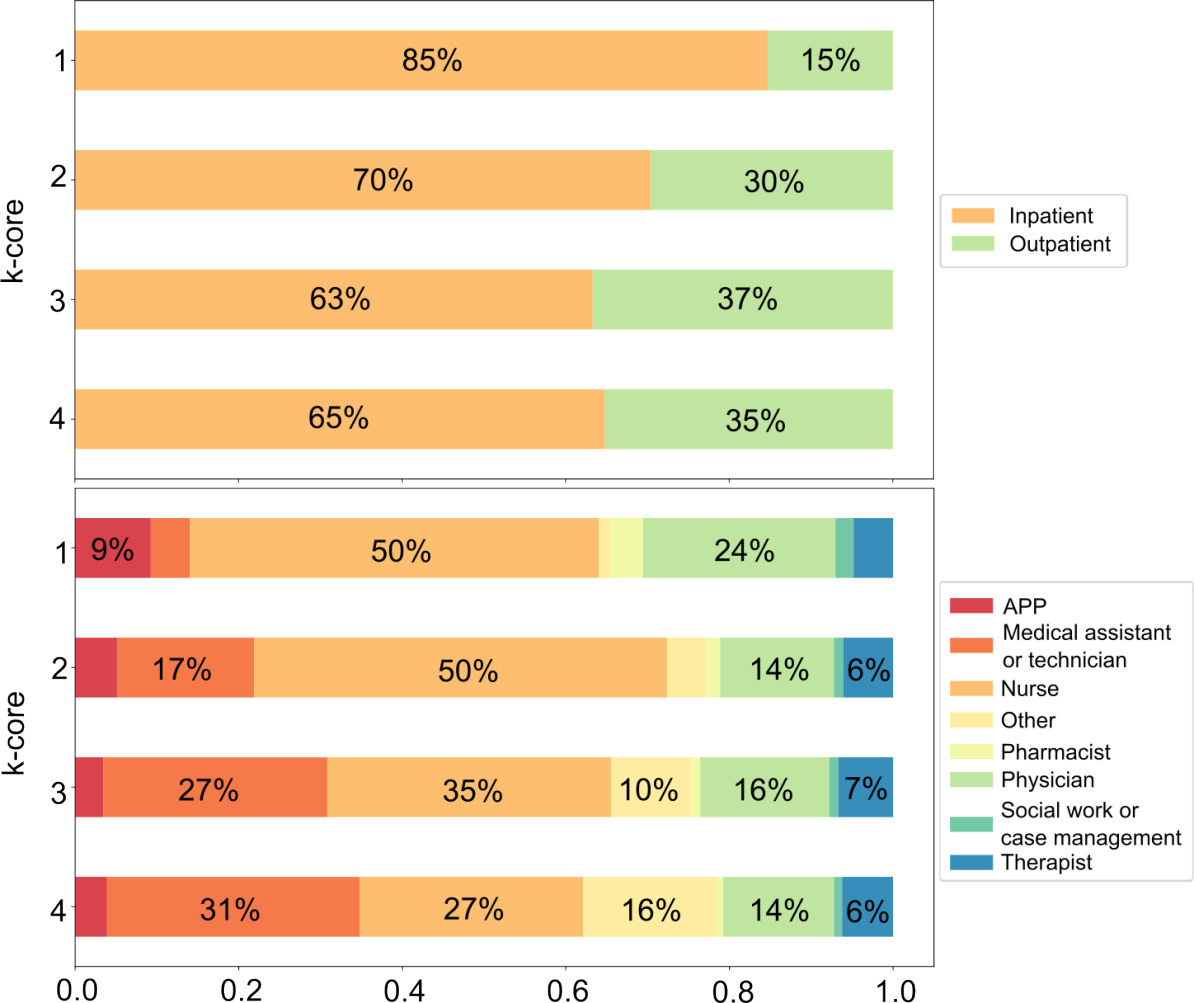
Distribution of users into the 4 k-core among each location and clinical role. Segments >5% were labeled with the percentage. APP: advanced practice provider.

The distribution of health care professionals into the 4 k-core layers was found to be significantly different between the practice locations (ie, inpatient and outpatient settings) (*P*<.001; Figure 1). The innermost central core had 30.4% (6423/21,133) of inpatient health care professionals as compared to 22.5% (4761/21,133) of inpatient health care professionals in the outermost peripheral core. Additionally, the innermost central core had 13.4% (1159/8662) of outpatient health care professionals as compared to 29.9% (2586/8662) of outpatient professionals in the outermost peripheral core.

#### Differences by Health Care Professional Role

There were statistically significant differences in all network metrics between health care professionals based on clinical role (*P*<.001 for all metrics; Table 3). Pharmacists and APPs had the highest values for all the network metrics, whereas medical assistants had the lowest values. For example, pharmacists had a median indegree of 47 (IQR 16‐116) and APPs had a median indegree of 36 (IQR 10‐107), while medical assistants had a median indegree of 7 (IQR 3‐17).

The distribution of health care professionals among the 4 k-core layers was significantly different across the health care professional roles (*P*<.001; Figure 1). There were 52.1% (290/557) of pharmacists in the innermost central core and 43% (701/1629) of APPs in the innermost central core, whereas 38.5% (2272/5907) of medical assistants were in the outermost peripheral core.

### Clusters and Messaging Behaviors

**Figure 2.**
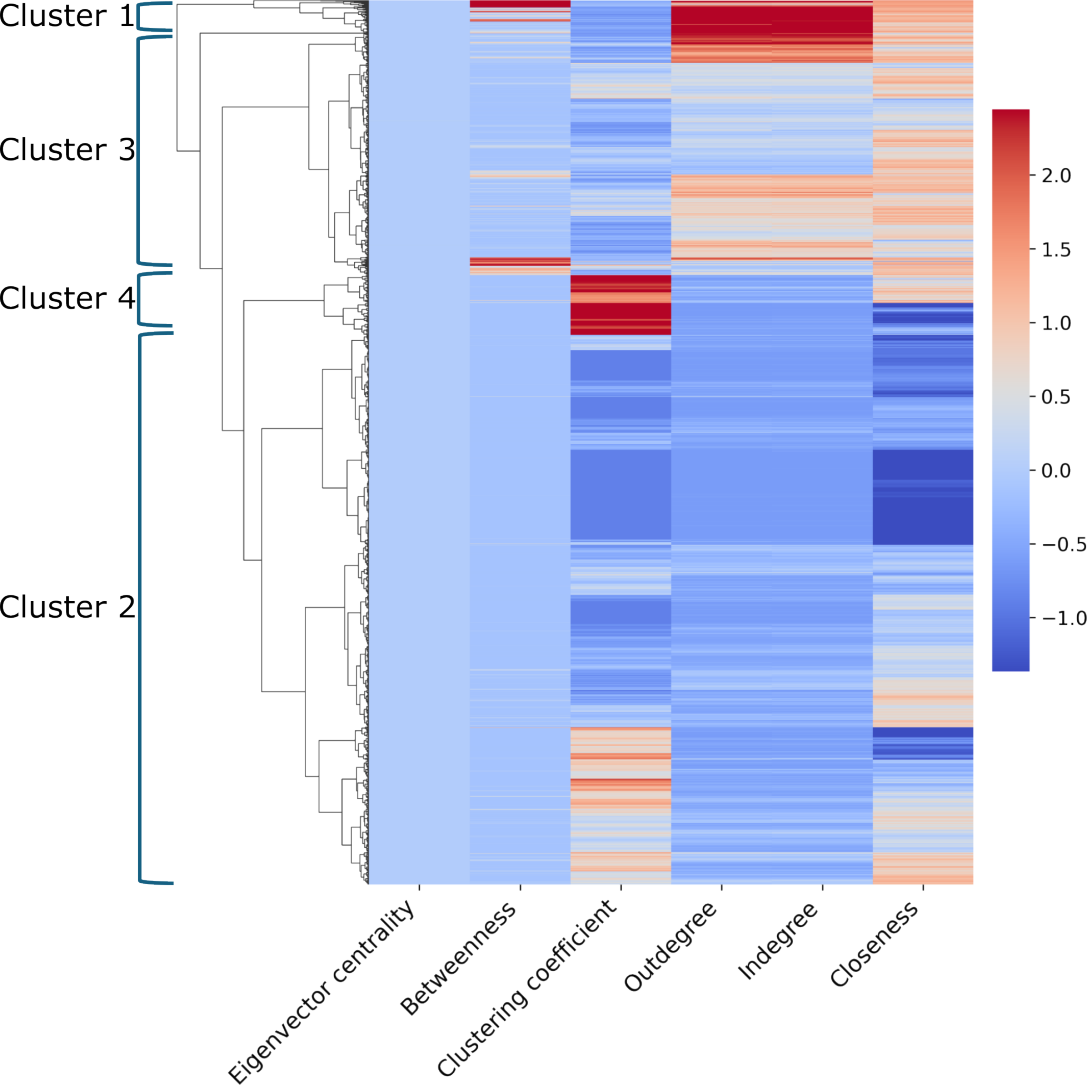
Heatmap for hierarchical clustering of health care professionals based on node-level metrics. The color bar depicts the magnitude of standardized values of the dataset, and the color range is computed with robust quantiles. The blue brackets indicate how clusters were divided into the final 4 groups with associated labels.

Hierarchical clustering on the node-level properties resulted in 4 clusters (see Table 4 and Figure 2). Cluster 1 had 82.2% (912/1109) of health care professionals working in an inpatient setting (Table 4). Furthermore, 59.2% (656/1109) of health care professionals in cluster 1 were physicians. The health care professionals in cluster 1 had the highest median values for the node-level metrics, except for the clustering coefficient, compared to the other groups.

**Figure 3.**
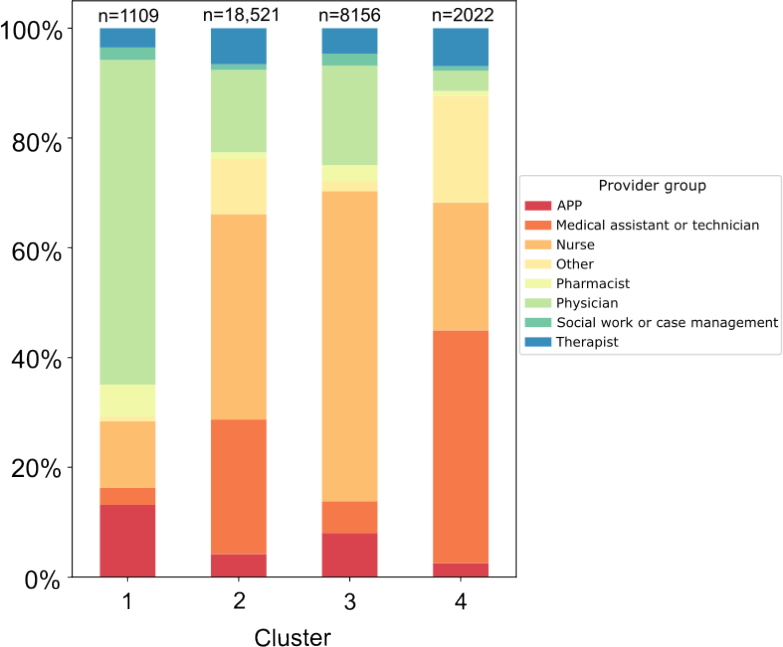
Composition of clinical roles within each cluster. APP: advanced practice provider.

Cluster 2 was the largest of the 4 identified clusters and included 62.1% (18,521/29,808) of the health care professionals from the study cohort. Cluster 2 had 68% (12,593/18,509) of health care professionals working in an inpatient setting. Furthermore, 37.4% (6933/18,521; Figure 3) of health care professionals in cluster 2 were nurses. The health care professionals in cluster 2 had the lowest median values for the node-level metrics compared to the other groups.

**Table 4.** Hierarchical cluster subgroups including the median (IQR) for the clustered node-level metric features and nonclustered secure message use features. Significance levels for pairwise comparisons with Bonferroni adjustment are indicated by letters a through c, as detailed in the footnote.

	Cluster 1	Cluster 2	Cluster 3	Cluster 4
Health care professionals, n	1109	18,521	8156	2022
Inpatient users, n/N (%)	912/1109 (82.2)	12,593/18,509 (68)	6674/8156 (81.8)	954/2021 (47.2)
Total messages sent over the study period, n	1,954,355	1,324,730	4,088,795	303,170
Total messages received over the study period, n	2,051,809	1,318,498	3,981,352	316,888
Eigenvector centrality, median (IQR)	4.934e-08 (1.473e-08 to 1.459e-07)	3.560e-11 (2.111e-12 to 5.647e-10)	3.927e-09 (4.942e-10 to 2.255e-08)	1.464e-11 (3.633e-13 to 1.896e-10)
Betweenness, median (IQR)	888,661 (233,794 to 3,700,421)	0 (0 to 3883)	64,866 (5270 to 269,634)	0 (0 to 3)
Clustering coefficient, median (IQR)	0.064 (0.051 to 0.087)	0.097 (0 to 0.244)	0.131 (0.082 to 0.194)	0.667 (0.6 to 1)
Outdegree, median (IQR)	265 (220 to 331)	8 (3 to 19)	71 (50 to 104)	6 (2 to 14)
Indegree, median (IQR)	264 (223 to 330)	9 (3 to 19)	70 (50 to 104)	6 (3 to 14)
Closeness, median (IQR)	266 (254 to 277)	180 (135 to 213)	244 (227 to 258)	186 (127 to 236)
Total message volume per day, median (IQR)	34 (25 to 48)	4 (3 to 7)^a^	12 (9 to 18)^a^^,^^b^	5 (3 to 8)^a^^,^^b^^,^^c^
Sent character length per message, median (IQR)	62 (48 to 80)	64 (44 to 93)	66 (52 to 85)^a^^,^^b^	49 (33 to 74)^a^^,^^b^^,^^c^
Received character length per message, median (IQR)	75 (63 to 83)	62 (45 to 86)^a^	61 (51 to 74)^a^	52 (39 to 73)^a^^,^^b^^,^^c^
Read time minutes, median (IQR)	14 (8 to 24)	22 (6 to 76)^a^	17 (9 to 33)^a^^,^^b^	26 (8 to 77)^a^^,^^c^
Response time minutes, median (IQR)	19 (13 to 30)	24 (8 to 71)^a^	27 (16 to 47)^a^^,^^b^	35 (8 to 107)^a^^,^^b^^,^^c^

a Indicates significant difference (adjusted *P*<.001) compared to group 1.

b Indicates significant difference (adjusted *P*<.001) compared to group 2.

c Indicates significant difference (adjusted *P*<.001) compared to group 3.

Cluster 3 had 81.8% (6674/8156) working in an inpatient setting. Furthermore, 56.5% (4608/8156) of health care professionals in cluster 3 were nurses. Cluster 4 had only 47.2% (954/2022) working in an inpatient setting. Additionally, 42.4% (858/2022) of health care professionals in cluster 4 were medical assistants. The health care professionals in cluster 4 had the highest median value for the clustering coefficient compared to other groups.

There were statistically significant differences in secure messaging behaviors across the 4 clusters (see Table 4, Multimedia Appendix 1; all *P*<.001 and footnotes indicate significant pairwise comparisons among the 4 groups). Compared to the largest subgroup (ie, cluster 2), cluster 1 had the quickest read (median –7.1, 95% CI –8.9 to –5.4 minutes) and response time to messages (median −3.2, 95% CI −4.9 to −1.7 minutes), the largest message volume per day (median 28.4, 95% CI 27.7‐29.2 messages), as well as the longest message character length (median 9.5, 95% CI 7.9‐11 characters) (see Table 4). Similarly, compared to cluster 2, cluster 4 health care professionals had slower response times (median 4.1, 95% CI 2.8‐5.6 minutes) and a marginally larger daily message volume (median 0.3, 95% CI 0.2‐0.4 messages), as well as sent (median −13.5, 95% CI −15.1 to −12 characters) and received (median −8.8, 95% CI −10.1 to −7.5 characters) the shortest messages.

## Discussion

### Principal Findings

Based on a large-scale study of over 31,000 health care professionals and more than 7.5 million secure messages, we found that the connectivity of the interactive communication network varied based on health care professionals’ roles and their practice settings. Specifically, inpatient health care professionals were generally more central and had the highest incoming and outgoing messaging connectivity compared to outpatient health care professionals, whereas pharmacists were the most central and highly connected among all the clinical roles. Hierarchical clustering analysis highlighted the differences in connectivity among the health care professionals and showed that cluster membership was associated with specific secure messaging behaviors such as read time, response time, total messaging volume per day, and character length per message. For example, cluster 1, which consisted of highly connected and central health care professionals, had the quickest read and response times to secure messages while also managing the largest message volume per day. Although preliminary, our findings highlight that an individual’s messaging behaviors are potentially driven by their networked peers. We describe some of the pragmatic implications of the current findings.

The degree of variation in connectivity between practice settings (ie, inpatient and outpatient) highlights the impact of setting-specific clinical context on resultant communication behaviors. For example, inpatient health care professionals, regardless of their role, had higher network connectivity in the communication network. The greater connectivity highlights their greater need for communication and care coordination, likely owing to the increased complexity of patient care delivery in these settings [49,50]. Ultimately, the setting-specific clinical context influences the extent of secure messaging need and burden.

The degree of variation in connectivity among clinical roles implies a potential relationship between the role-specific clinical context and the extent of secure messaging communication burden. For instance, pharmacists’ central position in the network identified them as key players in clinical communication and care management. This has previously been described in other studies that highlighted the central role of pharmacists in medication management and safety [51]. Additionally, in inpatient settings, pharmacists are often embedded within teams to help with coordinating complex medication management of patients, acting as gatekeepers for medication safety [52-54]. Thus, the unique communication needs among specific roles are apparent in the stark difference in secure messaging utilization and burden among professionals in our study.

The hierarchical clustering results revealed a relationship between connectivity within the network and secure messaging behaviors. For instance, health care professionals in cluster 1 were centrally located, highly connected, and acted as communication bridges (see Table 1, betweenness centrality, for definition) and were primarily consisted of physicians and those working in an inpatient setting. Health care professionals in this cluster also had the highest volume of secure messages per day, the longest character length per message received, and the fastest read times compared to the other clusters.

The higher centralization and connectivity in cluster 1 is likely because of their expertise and role, as the cluster is primarily composed of physicians (ie, as a clinical decision leader) [55]. Role-based specialization is common in teams where specialized individuals are relied on for specific expertise leading to targeted communication for specific tasks [55,56]. This is also reflected in cluster 1’s increased message volume and messaging behavior, most likely highlighting their role in clinical decision-making. However, such a centralized communication structure may also increase physicians’ workload and cognitive burden arising from an increased messaging volume [57].

Similarly, there was one cluster (cluster 4) of nurses and medical assistants who had fewer connections and were not as central within the network. These individuals were mostly involved in “communication cliques” with their immediately connected neighbors (ie, clinical colleagues) such that a given individual’s neighbors are also communicating with each other, forming close-knit circles in the network. The higher ratio of local clique structures for this cluster suggests increased mutual interaction and collaboration among health care professionals within a network [58,59]. Furthermore, cluster 4’s lower centrality scores (eg, eigenvector, betweenness, and closeness) suggest a more decentralized conversation structure, where communication is more evenly shared [55].

### Comparison to Prior Work

Understanding how secure messaging is being used can provide insights into communication and coordination patterns among health care professionals. More cohesive and collaborative networks of health care professionals have been shown to improve care quality for patients [23]. By understanding the state of collaborative interactions, we can potentially develop interventions targeted at improving these relationships and influencing downstream clinician and patient outcomes [26,27]. For example, network characteristics related to connectedness (eg, indegree) and structure (eg, clustering coefficient) can be used to identify “network traffic” and associated communication burden for specific health care professionals [51]. Such information about communication load can be used to develop “triaging strategies,” including creating messaging pools, redirecting messages, or assigning personnel for messaging-only roles [60-62]. These strategies have been shown previously to reduce email inbox message volume and self-reported time spent on inbox management [63-65]. Similar strategies could be developed to mitigate the messaging burden among health care professionals.

Similarly, as shown in our hierarchical cluster analysis, there were centrally located clinicians in certain clusters whose messaging behaviors were driven by their extant network. For example, we found that clinicians in cluster 1 were likely to have a higher volume of messages and more likely to read and respond to messages the quickest. Such messaging behaviors are likely to affect their clinical work activities, increasing their likelihood for increased workload, cognitive burden, and errors [10,57]. For example, Lew et al [57] found that inpatient clinicians receiving more messages spent more time on EHR-based work and had increased attention switching (ie, increased cognitive load); similarly, Lou et al [10] found that an increased volume of messages was associated with increased likelihood of wrong-patient ordering errors. The current findings point to the fact that increased messaging volume was potentially associated with their external connectivity, highlighting the role of connected communication partners (ie, network) in increasing workload, cognitive burden, and errors.

### Future Directions

Finally, it is important to emphasize that this was a preliminary study. More focused analyses (eg, among subgroups of clinicians such as hospitalists or outpatient clinicians) are necessary to ascertain specific strategies for bottlenecks in communication and improving clinician and patient outcomes.

### Limitations

There are several limitations in this study. First, although this study included several inpatient and outpatient settings across academic and community settings (14 hospitals, >250 outpatient locations), all of these were part of the same health care system using the only secure messaging platform available, Epic Secure Chat. Therefore, the generalizability of this study’s findings to other health care systems and other secure messaging platforms may be limited. Second, we only used one form of communication: secure messaging. Other forms of communication (ie, face-to-face, telephone, and pager) were also used by our cohort throughout the study period; therefore, our study likely underrepresents the true communication structures and relationships between health care professionals. Third, our assignment of clinical location (ie, inpatient vs outpatient) was based on log-in information and was potentially imprecise since we did not have access to detailed work schedules.

### Conclusions

This study characterized the network properties of a large-scale EHR-integrated secure messaging network using social network analyses. We found that health care professionals’ connectivity varied by both role and practice settings. Furthermore, hierarchical clustering indicated a relationship between an individual’s network connectivity and secure messaging behaviors. Ultimately, these preliminary findings highlight the importance of understanding secure messaging interactions and resultant secure messaging behaviors, as well as their potential impact on clinical work outcomes.

## Supplementary material

10.2196/66544 Multimedia Appendix 1 Additional figures and tables for the statistical analysis of secure messaging behaviors across cluster groups.

## Abbreviations

APP: advanced practice provider
EHR: electronic health record
GCC: giant connected component
IRB: institutional review board
