# Supplementary material for: Networked Behaviors Associated With a Large-Scale Secure Messaging Network: Cross-Sectional Secondary Data Analysis
Source: JMIR Med Inform. 2025 Jul 10;13:e66544. doi: 10.2196/66544 (PMC12287983; doi:10.2196/66544)
Supplement: Multimedia Appendix 1 [file medinform-v13-e66544-s001.docx]

**Figure S1.** Boxplots for each secure messaging behavioral outcomes among the 4 clusters. The significance was determined by the Kruskal-Wallis with a post hoc Wilcoxon Rank-Sum Test for pairs of cluster subgroups. Bonferroni correction was used for multiple comparisons. The significant codes indicate “***” <.001, “**” <.01, “*” <.05.


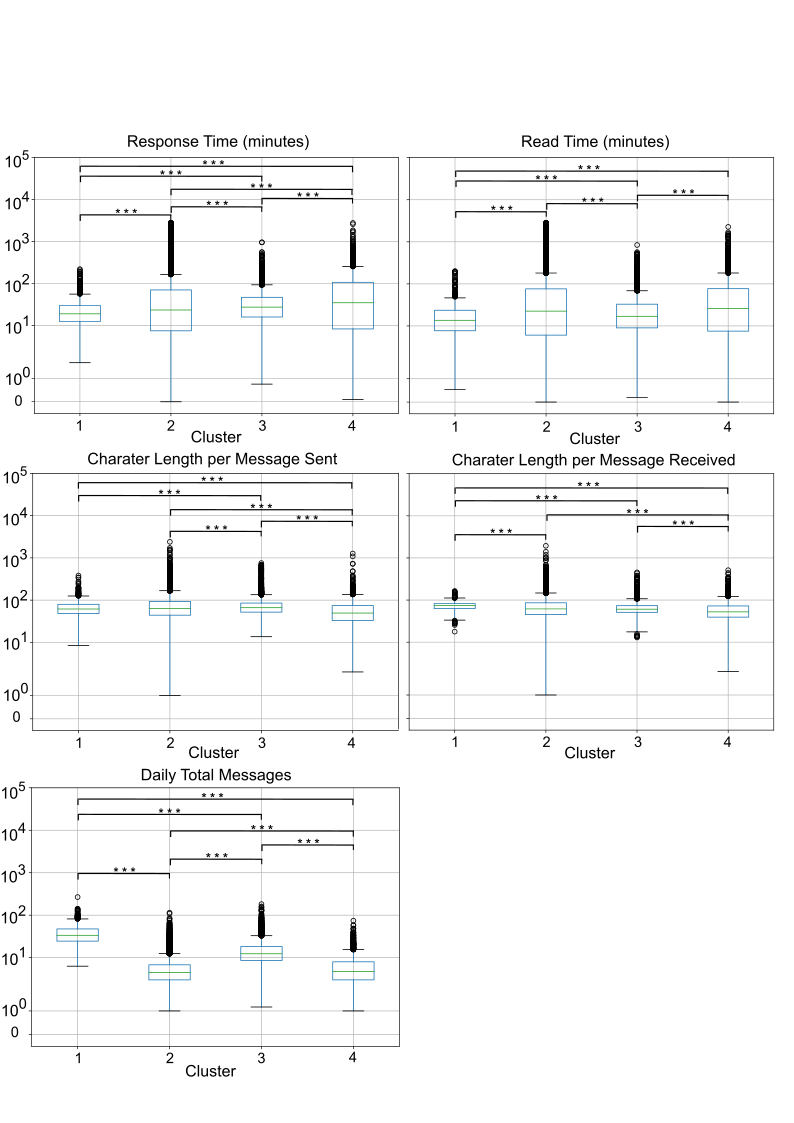


**Table S1.** Pairwise comparisons of the cluster subgroups for each secure messaging outcome. The significance was determined by the Kruskal-Wallis with a post hoc Wilcoxon test for pairs of cluster subgroups. Bonferroni correction was used for multiple comparisons.

| Secure messaging outcome | Pairwise comparison | Median | 95% CI | Adjusted *P* value |
| --- | --- | --- | --- | --- |
| Response time | 1 vs 2 | –3.2 | –4.8 to –1.7 | <.001 |
|  | 1 vs 3 | –7.0 | –8.1 to –6.0 | <.001 |
|  | 1 vs 4 | –12.3 | –16.0 to –8.9 | <.001 |
|  | 2 vs 3 | –3.0 | –3.6 to –2.4 | <.001 |
|  | 2 vs 4 | –4.1 | –5.6 to –2.8 | <.001 |
|  | 3 vs 4 | –4.2 | –6.3 to –2.3 | <.001 |
| Read time | 1 vs 2 | –7.1 | –8.9 to –5.4 | <.001 |
|  | 1 vs 3 | –3.0 | –3.8 to –2.3 | <.001 |
|  | 1 vs 4 | –10.3 | –12.6 to –8.2 | <.001 |
|  | 2 vs 3 | 3.4 | 2.7 to 4.1 | <.001 |
|  | 2 vs 4 | –1.1 | –2.1 to –0.1 | ns |
|  | 3 vs 4 | –6.2 | –7.6 to –4.8 | <.001 |
| Daily message volume | 1 vs 2 | 28.4 | 27.7 to 29.2 | <.001 |
|  | 1 vs 3 | 20.3 | 19.5 to 21.1 | <.001 |
|  | 1 vs 4 | 27.8 | 26.9 to 28.7 | <.001 |
|  | 2 vs 3 | –7.4 | –7.6 to –7.3 | <.001 |
|  | 2 vs 4 | –0.3 | –0.4 to –0.2 | <.001 |
|  | 3 vs 4 | 7.0 | 6.7 to 7.3 | <.001 |
| Sent character length | 1 vs 2 | –1.4 | –3.2 to 0.5 | ns |
|  | 1 vs 3 | –4.1 | –5.6 to –2.6 | <.001 |
|  | 1 vs 4 | 12.4 | 10.5 to 14.4 | <.001 |
|  | 2 vs 3 | –2.8 | –3.6 to –2.0 | <.001 |
|  | 2 vs 4 | 13.5 | 12.0 to 15.1 | <.001 |
|  | 3 vs 4 | 16.6 | 15.2 to 17.9 | <.001 |
| Received character length | 1 vs 2 | 9.5 | 7.9 to 11.0 | <.001 |
|  | 1 vs 3 | 11.1 | 10.0 to 12.2 | <.001 |
|  | 1 vs 4 | 18.6 | 16.9 to 20.2 | <.001 |
|  | 2 vs 3 | 0.5 | –0.2 to 1.1 | ns |
|  | 2 vs 4 | 8.8 | 7.5 to 10.1 | <.001 |
|  | 3 vs 4 | 8.3 | 7.3 to 9.3 | <.001 |
